# Navigation versus conventional high tibial osteotomy: systematic review

**DOI:** 10.1186/s40064-015-1040-5

**Published:** 2015-06-17

**Authors:** Khaled Hasan, Qusai Abdel Rahman, Paul Zalzal

**Affiliations:** Department of Orthopedic Surgery, McMaster University, 1280 Main St W, Hamilton, ON L8S 4L8 Canada; University of Toronto, 3359 Mississauga Rd, Mississauga, ON L5L 1C6 Canada

## Abstract

**Introduction:**

One major use for high tibial osteotomy aims at improving alignment in the symptomatic, varus malaligned, medial compartment osteoarthritic knee. The importance of achieving correct alignment is obvious upon considering the increased potential for significant complications with over- or undercorrection in any plane. The aim of this systematic review was to compare navigation and conventional high tibial osteotomy.

**Methods:**

This systematic review searched the MEDLINE and EMBASE databases to compare the short-term clinical and radiological outcomes between patients undergoing either conventional or navigated high tibial osteotomy.

**Results:**

We retrieved 71 articles, which ultimately resulted in 14 eligible studies for inclusion. Though heterogeneity prevented statistical analysis, only one study failed to suggest superiority of navigation over conventional techniques.

**Conclusion:**

Navigated high tibial osteotomy improves accuracy over conventional techniques, though the current best evidence presented herein must be advanced by higher quality studies.

**Electronic supplementary material:**

The online version of this article (doi:10.1186/s40064-015-1040-5) contains supplementary material, which is available to authorized users.

## Background

High tibial osteotomy (HTO) is a procedure designed to change the alignment of the knee. It is indicated for the treatment of symptomatic medial compartment osteoarthritis in varus malaligned knees as well as patients with osteonecrosis in the medial condyle. The HTO procedure results in significant pain relief and restoration of function as the weight-bearing load is transferred to the intact compartment of the knee to alleviate symptoms, slow disease progression and defer subsequent total knee arthroplasty (Kim et al. 2009). In fact a study using bone densitometry has demonstrated changes in loading distribution before and after HTO (Agneskirchner et al. 2004). A known difficulty with the procedure is demonstrated intraobserver variability and low reproducibility, even with regards to coronal plane alignment correction (Coventry 1979; Cullu et al. 2005; Engel and Lippert 1981; Hernigou et al. 1987). Thus, conventional HTO technique has shown a high degree of alignment variability when measuring post-operative alignment (Agneskirchner et al. 2004; Bae et al. 2009; Coventry 1979; Cullu et al. 2005; Engel and Lippert 1981; Hernigou et al. 1987). It is essential that the correct alignment be achieved with the HTO as under- or overcorrection can have significant mechanical complications. In addition, multi-planar deformity may be present and can be either under or overcorrected during the procedure. While undercorrection leads to progression of medial joint arthritis and patient dissatisfaction, overcorrection leads to patellar subluxation, patella baja, medial joint opening and rapid degeneration of the lateral cartilage (Agneskirchner et al. 2004; Billings et al. 2000; Brouwer et al. 2005; Chae et al. 2008; Coventry 1979; Coventry et al. 1993; Flecher et al. 2006). Thus, to prevent overcorrection and undercorrection in HTO, the desired correction angle should be calculated exactly in preoperative planning (Kim et al. 2009). HTO may change the tibial slope in the sagittal plane, altering the tension of the anterior and posterior cruciate ligaments (Gebhard et al. 2011; Gunes et al. 2007). Also, an increase in tibial slope increases anterior–posterior instability of the knee and may lead to increased contact pressure in the posterior femorotibial compartment in the case of ACL deficiency (Coventry and Bowman 1982; Cullu et al. 2005; Fujisawa et al. 1979).

Navigation, therefore, could become an important device for intra-operative control, and early literature reported encouraging results (Agneskirchner et al. 2004; Bae et al. 2009; Engel and Lippert 1981; Fujisawa et al. 1979) given that computer-assisted operation have gained popularity in orthopedic surgeries as they can improve intraoperative accuracy, precision and reproducibility (Kim et al. 2009). Veritably, recent HTO computer-assisted technique was shown to be capable of accurately measuring leg alignment intra-operatively with especially high precision in the coronal plane (Gebhard et al. 2011; Giffin et al. 2004). Furthermore, it allows a 3D display of the limb compared to the 2D radiographs of conventional HTO methods (Ribeiro et al. 2013). The purpose of this study was to compare short-term clinical and radiological results between two groups of patients treated by HTO utilizing either standard technique, such as the rule of thumb, the trigonometric principle and the weight-bearing line method (Kim et al. 2009), or navigated technique. This study assessed postoperative mechanical axis alignment outcomes.

Medial opening wedge HTO has the advantage of acquiring accurate post-operative alignment and therefore, we focused in our review to include studies that only included such technique. Postoperative alignment of the lower limb is crucial for success in opening wedge HTO, although preservation of post-operative alignment in the long term remains uncertain. Early post-operative alignment in opening wedge HTO depends on an accurate pre-operative planning and an accurate intra-operative technique.

Computer navigation systems for total knee arthroplasty have facilitated more accurate correction of alignment, and so, it is hypothesised that navigation would provide more accurate alignment correction in HTO as well. To answer this question, a systematic review was conducted to look at whether conventional versus navigated HTO provides a more accurate correction of alignment.

## Methods

This systematic review adhered to the reporting guidelines of the Meta-analysis of Observational Studies in Epidemiology statement (Stroup et al. 2000).

### Eligibility criteria

The authors identified articles in English that met the following eligibility criteria: (1) the study was published within the last 20 years and (2) the study compared computer-assisted HTO results with conventional HTO for opening wedge osteotomies, as closing wedge osteotomies were not of particular importance in this study. Studies were excluded for the following reasons: (1) no comparison of computer-assisted versus conventional HTO’s and (2) the article did not provide direct measurements of immediate post-operative results following HTO.

### Identification of studies

The authors conducted a search of MEDLINE and EMBASE for relevant articles published within 20 years in the English language. The following key words were used: HTO or computer-assisted surgery or computer-assisted technique or navigation or navigated and conventional or medial opening wedge. To identify any studies that we did not capture with our MEDLINE search, we consulted a content expert and reviewed reference lists from articles that fulfilled their eligibility criteria.

### Assessment of study eligibility

Two of the authors independently assessed the studies identified for full evaluation and resolved disagreements through discussion until consensus was reached. One author additionally verified inclusion of all studies.

### Assessment of methodological quality

Four reviewers independently graded the methodological quality using the Newcastle Ottawa Scale of included studies, with two reviewers grading each paper (Wells et al. 2015).

### Data extraction

Two authors developed a structured data extraction form and extracted data from each eligible study. This structured form ensured that all data were being extracted and recorded consistently and completely by all reviewers. They extracted data on study characteristics, including location of study, measurements of correction angles, method of data collection, etc. We extracted data directly comparing accuracy of correction of navigated HTO versus the conventional procedure.

### Data analysis

The authors collected data comparing accuracy of HTO measurement results using navigation versus conventional from the included studies. We also compared HTO accuracy with navigation across studies to determine which method provided more accurate correction in HTO, navigation or conventional approaches.

### Pre-analysis hypothesis

Before analysing the data, we hypothesised that the majority of the studies would show that navigated HTO would provide more accurate correction in comparison to conventional techniques.

## Results

### Study identification

**Figure 1:**
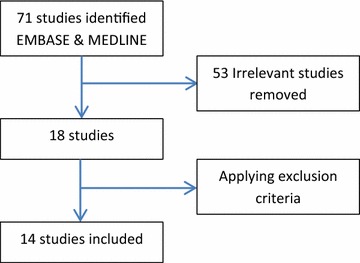
Schematic representation of literature search.

The authors identified 71 articles through an EMBASE and MEDLINE search that were possibly relevant, 53 of which were excluded after review of titles, either because they were irrelevant to the subject matter or because they were not in English, and thereby did not meet the inclusion criteria. An additional four studies were excluded after review of abstracts (Figure 1). Thus a total of 14 studies were included in this review from all examined sources.

### Study characteristics

Fourteen studies, published between 2005 and 2014, met the aforementioned inclusion criteria for this review (Additional file 1: Table S1). Thus, our sources constitute fairly recent data and the latest best evidence. However, no prospective randomised control trials comparing navigated versus conventional HTO with respect to obtaining better postoperative mechanical axis and posterior tibial slope alignments exist. Three studies included herein were performed on cadavers, with the remainder being mostly Level 3 in quality, with one Level 4 and one Level 2 prospective cohort study. The overall quality is attributable, in part, to the relatively small sample sizes of each study as most of the studies included less than 56 subjects, except for Kim et al. (2009) work in which 85 subjects were included in the study. Though age ranges, gender splits, and knees operated on (left versus right) were not reported in all studies, general age ranges included from 35 to 76 years. The majority of studies had more male patients included, with two studies from Korea having females comprise approximately 77 and 92% of the study population. On average, of those studies reporting right versus left knee, 54% of the knees examined were on the right. The heterogeneity of the studies, from subject gender and type (human versus cadaver), osteotomy technique (open versus closed-wedge), navigation system used, and follow-up periods, to name a few, prevented data assembly for statistical analysis. Despite an extremely meticulous search of the literature with broad search terms as the foundation, including only English studies herein may also contribute to our findings. In fact, the studies analysed showed a wide variety in terms of location of publication, as 62% of included studies were conducted in Europe (majority Germany), 23% in Asia and 15% in Latin America (Brazil). Taking this and the implied differences in surgical technique, study designs, and so on that are inevitable, together with the current controversy regarding the ideal correction angles, affords merely identifying trends in the data at best.

Findings of included studies were extracted and arranged in a tabular form (Additional file 2: Table S2). Thus describing the surgical techniques applied in HTO, the study outcomes and the conclusion reached by each study.

## Discussion

Navigation systems are exacting an ever-increasing presence across all surgical disciplines, most notably, orthopaedics. Promising increased technical precision, reduced operating time, decreased radiation exposure and ultimately, enhanced patient safety as surgical navigation is projected to experience moderate growth through 2016, at least in the United States. As these systems and associated software present a financial constraint within some health care systems, justifying their superiority over conventional methods is paramount. In orthopaedics, the implementation of navigation and its effects on more precise limb alignment, and subsequently better clinical outcomes in total knee arthroplasty cases is well documented. As such, this systematic review aims at elucidating whether or not the benefits of navigation are analogous in patients undergoing HTO for medial compartment, osteoarthritic, varus malaligned knees. It is our hypothesis that navigation does in fact offer enhanced radiological accuracy with regards to lower limb alignment than the conventional technique. Specifically, we expect computer-assisted HTO to be superior to the standard techniques with regards to achieving a post-operative mechanical axis between 2° and 6° valgus and ±2° in posterior tibial slope. Within these parameters, navigated HTO should improve clinical outcomes via limiting under-or overcorrection of mechanical axes common with the standard technique. Upon completing a thorough search of the existing literature, this review aims to be the first to compare clinical and radiological outcomes between patients undergoing HTO via navigated or standard avenues (Additional file 3: Table S3).

Certainly in cadaver studies, navigation has proven tremendously precise. Navigation has demonstrated correction of mechanical axes to within 0.5°–0.7° (Lutzner et al. 2009). Other measures, aim to align the mechanical axis at the 80% width mark of the tibial plateau, with 0% representing the medial edge and 100% the lateral edge. Cadaver studies further demonstrate significance (P < 0.02) with navigation, as such techniques not only established a mechanical access at the 79.7% (range 75.7–85.8%) mark versus 72.1% (range 60.4–82.4%) range conventionally, but did so with significantly less variability (Hankemeier et al. 2006). These findings are consistent in human studies as well, with one study having all navigated cases and only 55% of conventional cases within the tolerable (±1° or within ±5%) range for intersection at the 80% width mark (Lutzner et al. 2010). In this study, the error was ±1% in navigated and ±8.6% (P = 0.002) in the conventional cases, a finding echoed by the work of Gebhard et al. (2011) showing 85% (and likely as high as 93%) accuracy in the final post-operative correction as compared to initial pre-operative plans.

Iorio et al. (2013) also suggest superiority with navigation systems over conventional means, demonstrating significant deformity correction in 86% of computer-assisted cases versus 23% conventionally (P = 0.0392). As deformity correction is multi-planar, navigation also appears superior to standard techniques in attaining a desired 170° (±3°) coronal femoral tibial angle, and posterior tibial slope angle of +2° to −2° (Akamatsu et al. 2012; Iorio et al. 2013). Every navigated case versus 24% of conventional cases achieved target corrections for the posterior tibial slope (P = 0.0021) in the Iorio et al. (2013) work, with these findings parallel to that of Akamatsu et al. (2012) and Bae et al. (2009) with statistical significance. Navigation platforms were consistently within normal ranges (P = 0.001), agreeable with post-operative radiographs (p < 0.05), and showing less variability (P < 0.012) than its conventional counterpart (Akamatsu et al. 2012; Bae et al. 2009).

Moreover, in the Ribeiro et al. (2013) work it was noted that the navigation system allows a 3D evaluation of the limb, while radiographs provide only 2D images, thus better representation of the knee and greater accuracy. In fact the calculated mean opening obtained by the Dugdale et al. method in that paper was 9.53° and 11.8° by the navigation system measurement, which was a statistically significant difference (P = 0.0359 by Student’s t test and P = 0.045 by Kruskal–Wallis test). Ribeiro et al. also points out the importance of increasing the accuracy upon HTO procedure, through reduction of the human error involved upon performing the surgery. Mistakes in measurements with a ruler and pencil associate more error into the surgery than using the digital radiography that enables more accurate measurements (Ribeiro et al. 2013). Such error is followed by amplified consequences as undercorrection of the leg axis causes an incomplete transfer of weight from the medial to the lateral compartment, whereas overcorrection causes the knee to become unstable allowing arthritis to progress faster in the lateral compartment (Ribeiro et al. 2013).

More recent work of Ribeiro et al. (2014) further emphasize on the significant better control of tibial slope and higher Lysholm score using navigation system in a different study; as after surgery the control group presented tibial slope of 13.75 ± 3.75 and Lysholm score of 87.60 ± 11.12 compared to tibial slope of 10.11 ± 0.18 and Lysholm score of 91.94 ± 11.61 in the navigated group. Upon comparing the results of the study, it can be noticed that there is a significant decrease in the tibial slope when the navigation procedure was used, as well as a significant increase in the Lysholm score. Both of these findings contribute to the desired results and thus provide further support for the navigated approach as it shows greater accuracy (Ribeiro et al. 2013).

Concerning retrospective studies, the work of both Kim et al. (2009) and Maurer et al. (2006) favored the navigated HTO over conventional HTO. In which Maurer et al.’s (2006) results showed a significantly higher accuracy in achieving the proper leg axis correction (P < 0.016) when HTO was performed with navigation. Furthermore, on radiographic assessment of Kim et al.’s (2009) work the navigation group showed better results than the conventional group in both mechanical axis and the coordinate of the weight bearing line on a full-length standing anteroposterior radiograph (3.9° ± 1.0° vs. 2.7° ± 2.2° of valgus, P < 0.01), (62.3 ± 2.9 vs. 58.7 ± 6.6% coordinate at the tibial plateau, P < 0.01). Moreover, on clinical assessment of the same paper, the navigation group showed better results in both the mean Hospital for Special Surgery knee score (84 ± 8 vs. 79 ± 7, P < 0.01) and the mean Lysholm knee score (85 ± 6 vs. 83 ± 5, P < 0.05). As with the cohort study by Saragaglia et al. (2005) results have shown 96% reproducibility in achieving mechanical axis of 184° ± 2° in the navigated group compared to 71% reproducibility in achieving the same mechanical axis of 184° ± 2° in the conventional osteotomy group (P < 0.0015).

Lee et al. (2012), examining the utility of intra-operative navigation, echoes the current literature, demonstrating significant improvements in degree of osteoarthritis (P < 0.015) and knee ligament condition (P < 0.032), in addition to suggesting navigation reaches mechanical axis targets (84.6% of knees) and weight-bearing length ratio targets (74.4% of knees) in patients studied. However, their measurements correlated only to a ‘fair’ degree with post-operative films, as they were made under non-weight bearing conditions, and were limited to only the coronal plane. Therefore, generalisations of navigation for improving total lower limb alignment from the Lee et al. (2012) study cannot be made. Of note, only one study demonstrated no significant difference in correction between computer-assisted and standard approaches, yet still noted that all navigations were consistent with pre-planned ranges, which is not always the case when applying standard techniques (Reising et al. 2013).

Interestingly, navigation alone does not affect operating time in any meaningful manner, as the Reising et al. (2013) study had an average time of 141 min between both groups, and Gebhard et al. (2011) documented average operating time of 105 min, with 60% of procedures completed within 60–120 min. In fact, Akamatsu (2012) noted that total operative time was significantly longer in the navigated group than in the conventional group (P < 0.001), whereas Hankemeier et al. (2006) suggested navigation having 23 less minutes of operative time, and less fluroscopic time (P < 0.001). Thus, no solid conclusion can be made concerning the change in operational time if navigated HTO was performed.

Moreover, success with navigation does not appear to be dependent on experience with the system, as both experienced and navigation-naive practitioners demonstrated good inter- and intra-observer reliability (Lutzner et al. 2009). Navigation is also unlikely to cause significant postoperative complications (Lutzner et al. 2009). In Ribeiro et al. (2014) study, it was noted that after few years of the surgery there is a natural tendency to progressive loss of correction obtained and consequently deterioration of the clinical improvements, but the use of navigation adds precision to the amount of correction needed and maybe represents a gain of time to the treatment of the affected knees (Ribeiro et al. 2014). The study however emphasize on the importance of long term follow up visits, and thus clinically so far appears to be no advantage of navigation over conventional techniques (Akamatsu et al. 2012; Iorio et al. 2013). This is attributable to inadequate follow-up data and insufficient sample sizes. As such, in light of the well-accepted premise that avoidance of under or overcorrection leads to better function, the current best evidence would suggest use of navigation to ensure optimal radio-graphic alignment, and in turn, outcome. Ideally, prospective randomised clinical studies with long-term follow-up, of which none currently exist, are fundamental to ultimately defining the role of navigation in HTO.

## Conclusion

HTO remains a viable option for patients with isolated medial compartment knee arthritis. Accuracy of correction of alignment is a crucial factor in determining patient outcome. With the importance of achieving an accurate correction it appears upon comparing computer-navigated HTO to conventional HTO that navigated HTO provides more accurate corrections, thus reduces the potential consequences that can follow due to over or undercorrection such as predisposing to malalignment, pain, and arthritis. The vast majority of the studies used in this literature indicate the superiority of the navigation HTO due to significant reduction in the error involved upon taking pre-operational measurements as hypothesized. In addition, upon follow up, most studies showed significant difference and improvement in results. However, more studies and high-quality trials need to be conducted, as the literature on this topic remains sparse.

## Additional files

Additional file 1:
**Table S1.** Characteristics of included studies. Additional file 2:
**Table S2.** Findings of included studies. Additional file 3:
**Table S3.** Rating system for the hierarchy of evidence (Melnyk 2005).
