# Supplementary material for: Navigation versus conventional high tibial osteotomy: systematic review
Source: Springerplus. 2015 Jun 17;4:271. doi: 10.1186/s40064-015-1040-5 (PMC4469593; doi:10.1186/s40064-015-1040-5)
Supplement: Additional file 1: — Table S1. Characteristics of included studies. [file 40064_2015_1040_MOESM1_ESM.docx]

| **Table S1 Characteristics of included studies** | | | | | | | | |
| --- | --- | --- | --- | --- | --- | --- | --- | --- |
| **Study** | **Country** | **Years Examined** | **Indications** | **Patients** | **Knees** | **Follow-Up** | **Type** | **Evidence Level** |
| Iorio et al.  2011 | Italy | Not  reported | -varus deformity  -symptomatic medial compartment OA  -age < 65, grade 3 or lower Kellgren-Lawrence, failed  -conservative management, absence of additional  -cartilage procedures and concomitant ligamentous lesions | T: 24  C: 11  N: 13 | T: 27  C: 13 (7 male,  R/L not noted)  N: 14 (7 male,  10 right knees) | 39  months  (mean) | Therapeutic  Study,  Prospective  Cohort | 2 |
| Akamatsu  et al. 2011 | Japan | C: 2003-2006  N: 2006-2009 | C: 22 knees primary OA, 6 knees  osteonecrosis  N: 31 knees primary OA, 9 knees  osteonecrosis | C: 22  N: 26 | C: 28 (13 right)  N: 31 (17 right) | 1 year  post-op | Retrospective | 3 |
| Reising  et al. 2012 | Germany | C: 2005-2009  N: 2005-2009 | -medial varus OA | C: 40 (age ~43,  32 male)  N: 40 (age ~43,  32 male) | Not Reported | Not Reported | Retrospective  Case-Control | 3 |
| Bae  et al. 2011 | Korea | C: Jan. 1994-  July 2006  N: July 2005-  July 2006 | C: medial compartment OA : genu varum (43:7)  N: medial compartment OA : genu varum (48:2) | C: 34 (age 45-76,31 female)  N: 48 (age 48-71, 44 female) | C: 50 (30 right)  N: 50 (26 right) | Not  Reported | Matched  Retrospective  Cohort | 3 |
| Ribeiro  et al. 2013 | Brazil | Aug 2010 – May 2011 | -Varus deformity  -Medial compartment osteoarthritis | 17 patients (age 24-59, mean 45.7, one female) | 8 Right Knee,  9 Left Knee | 12 weeks | Retrospective | 3 |
| Kim et al. 2009 | South  Korea | April 2004 – December 2007 | - Medial unicompartmental osteoarthritis with varus deformity  -activity related pain on the medial side of the knee  -flexion arc of more than 100˚with less than 15˚ of flexion contracture  -less than 12˚ of varus malalignment | 85 patients | 90 Knees,  C: 43, 38 male  N: 47, 43 male  R/L not noted  in both C and N | 1 year | Retrospective comparative study | 3 |
| Maurer  et al. 2006 | Germany | January 2003 – March 2006 | -Genu valgum | 44 patients  (50-70 years old)  (33 females) | 67 knees  (C: 23, N: 44)  (41 right, 26 left) | Not reported | Retrospective comparative study | 3 |
| Saragaglia  et al. 2005 | France | Group A March 2001 – April 2002  Group B January 1997 – December 2000 | -Osteoarthritis secondary to genu  varum  -Osteoarthritis stage 1, 2 and 3. | 170 Patients  Group A, mean: 54 range:35-71  Group B,  mean: 55  range:27-70 | 170  Knees (135  for HTO)  (From which 28 were randomly selected for navigation and another 28 for conventional) | Not reported | Cohort study | 3 |
| Ribeiro  et al. 2014 | Brazil | 2004-2012 | Idiopathic unicompartmental medial osteoarthritis | 38 patients | 38 knees  C:20 (12 male,  11 right)  N:18 (17 male,  9 right) | 1 year | Retrospective Comparative study | 3 |
| Gebhard  et al. 2009 | Germany | Jan. 2006-  Oct. 2007 | -medial gonarthritis or genu  varumcongenitum  -age > 18  -42/51 subjects had prior surgery  on aﬀected leg  -39/51 subjects had a congenital  deformity vs post-traumatic | N: 51 (40 male) | N: 51 (23 right) | 6 weeks  (98%  follow-up) | Prospective  Multi center  Study,  Case Series | 4 |
| Lutzner  et al. 2010 | Germany | 2008-2010 | -not reported, 3 legs excluded for  serious restrictions with hip and  knee ROM | 11 human cadavers | C: 9 legs  N: 10 legs | N/A | Cadaver  Study | 5 |
| Hankemeier  et al.  2006 | Germany | Not reported | -no speciﬁc pre-op indications  -excluded if previous TKA or  osteosynthesis of tibial head | 11 patients (age 35-71,  7 male) | 20 cadaver legs  C: 10 legs  N: 10 legs | N/A | Cadaver  Study | 5 |
| Lutzner  et al. 2009 | Germany | 2008-2009 | -mechanical leg axis 4 degree  varus | 1 leg, 4 surgeons, 5  assessments per surgeon | 1 leg | N/A | Cadaver  Study | 5 |
