# Supplementary material for: Navigation versus conventional high tibial osteotomy: systematic review
Source: Springerplus. 2015 Jun 17;4:271. doi: 10.1186/s40064-015-1040-5 (PMC4469593; doi:10.1186/s40064-015-1040-5)
Supplement: Additional file 2: — Table S2. Findings of included studies. [file 40064_2015_1040_MOESM2_ESM.docx]

| **Table S2 Finding of included studies** | | | |
| --- | --- | --- | --- |
| **Study** | **Osteotomy techniques and evaluation method** | **Study Outcomes** | **Concluding statement** |
| Iorio et al. 2011 | - Kinematic data including hip, ankle, and knee joints were registered using OrthoPilot along with necessary anatomic landmarks for preoperative planning.   - A standard longitudinal incision was made medial to the patellar tendon to expose the proximal and medial tibia subperiosteal. The osteotomy began 3 cm distal to the medial joint line at the medial cortex of the proximal tibia and was proximal to tibial tubercle.   - By monitoring the mechanical leg axis and changing the tibial slope with the use of 3D navigator, the osteotomy was stabilized using (Position HTO Plate) with rectangular spacer block varying in size according to the degree of correction. | - Upon 39 month follow up, radiological results showed 86% reproducibility in achieving mechanical axis of 182° - 186° in the navigated group compared to 23% in the conventional group ( p = 0.0392).  - A modification of posterior tibial slope between -2° and +2° was achieved in 100% of the navigational group patients, whereas in the conventional group, this goal was achieved only in 24% of the cases. | - High tibial osteotomy with navigator is more accurate and reproducible in the correction of the deformity compared to standard technique. |
| Akamatsu et al. 2011 | - In conventional group, after incision the oblique line of the osteotomy was drawn at a position from 30 to 35 mm distal to the medial tibial joint line to 10–15 mm distal to the lateral tibial joint line.   - Under the image intensifier, the osteotomy line was gradually opened to the axis on the lateral cortex by stepwise insertion of 3–5 coupled chisels. Two wedges adjusted to the size of the osteotomy gap were inserted.  - In navigation group, OrthPilot was used to determine correction angle and register anatomical landmarks and thus the system created an adopted knee bone model. | - Mean postoperative Femoro-tibial angle (FTA) was higher in conventional group than in the navigated group (p = 0.037).  - 4 lateral unstable knees (knees with lateral cortex breakage or lateral tibial plateau fracture) were corrected to 174.6° in the conventional group, and 5 lateral unstable knees in the navigated group were corrected to 170.3° (none of which showed FTA greater than 173°)  - Mean change in tibial posterior slope (TPS) was greater in the conventional group than in the navigated group (p = 0.001). | - The navigated system in opening wedge HTO might reduce undercorrection in the knees with lateral cortex breakage or lateral tibial plateau fracture, and provide the better intraoperative (FTA) and (TPS). |
| Reising et al. 2012 | - The mechanical axis was shifted to cross the lateral aspect of the tibial plateau at 62 % on the cross sectional diameter and the osteotomy was stabilized with the Synthes TomoFix instrument.  - At the start of surgery, the navigation pins were anchored in the region of the distal femur or proximal tibia using 4.5 mm screws at an angle of 90° to the diaphyseal axis and 30°–45° from the medial side to the vertical axis allowing the Orthopilot software to register and identify anatomical landmarks of the knee.  - Limb alignment of the affected leg was assessed using the technique of Pauwels, according to this technique the limb alignment was taken as connecting line through the center of the hip, knee and ankle. | - As a percentage of the width of the tibial plateau the postoperative weight-bearing radiographs showed a mechanical line that intersected with the knee base line at the desired value of 62% (Fujisawa point) in 58.8 % (SD±6.1) in navigational group and in 58.6% (SD±8.1) in conventional group.  - Despite similar mean values a significantly higher number of corrections were outside the reference area (n = 7) in the conventional group, whereby all corrections were within the desired range in the navigated group.  - There were no significant differences in operation time. | - The use of a navigation system cannot increase the precision of the open-wedge HTO procedure in patients with varus osteoarthritis but it can eliminate the outliers of a well-defined range. |
| Bae et al. 2011 | - Following incision in navigated group, joint capsule and articular surface between the fibular head and proximal tibia were removed using osteotome and a pituitary rongeur.  - 2 reference bases were fixed to the distal femur and the middle tibia with a pair of half-pins, allowing kinematic and anatomical registration of 8 landmarks.   - The software sets the cut plane of the osteotomy perpendicular to the mechanical axis according to the sagittal plane; optimum correction of the axis at 62%.  - Using a navigated drill guide 2 Kirschner (K)-wires were placed in the proximal plane of the osteotomy and 2 in the distal plane. An electric saw was used to carry out proximal and distal osteotomy over the two K-wires. | - In the navigation group, the mean mechanical axis prior to osteotomy was varus 8.2°, and the mean mechanical axis following fixation was valgus 3.6°. On the radiographs the mean preoperative mechanical axis was varus 7.3°, and the mean post-operative mechanical axis was valgus 2.1°.  - There was a positive correlation between the measured data taken under navigation and by radiographs (r > 0.3, p < 0.05). The mean correction angle was significantly more accurate in the navigation group (p < 0.002). The variability of the correction was significantly lower in the navigation group (2.3° *vs* 3.7°, p = 0,012). | - The navigation provides reliable real-time intra-operative information, may increase accuracy, and improves the precision of a closed-wedge HTO. |
| Ribeiro et al. 2013 | - All patients underwent panoramic radiography with bipedal load for the wedge calculation by Dugdale method (DM). They underwent HTO with an opening wedge, fixed with an HTO plate and monitored by the OrthoPilot Navigation System (NS).  - Bone grafts were used in every case. The wedge opening obtained by the NS was compared to that calculated in the radiographs. | - The mean opening obtained, calculated by the Dugdale et al. method, was 9.53° and 11.8° by the navigation system measurements.  - This difference was statistically significant (p = 0.0359 by Student’s t test and p = 0.045 by Kruskal-Wallis test). | - There was a significant difference in the calculation of the wedge opening between the DM and NS. HTO without the aid of the NS could theoretically lead to undercorrection of the deformity. |
| Kim et al. 2009 | - Medial open wedge high tibial osteotomy was employed for both groups, except that in the navigation group, the reference markers for determining the mechanical axis were used in the setting of the kinematic navigation system (Orthopilot) and an allogenous chip bone graft was used to reduce overall operation time.  - In the conventional group, the cable method was used by drawing one line from the center of the hip to the 62% coordinate on the tibial plateau and a second line from the 62% coordinate on the tibial plateau to the center of the ankle joint, and thus determines the expected correction.  - The autogenous tricortical iliac bone graft was also used to enhance union. | - On radiographic assessment, the navigation group showed better results than the conventional group in both the mechanical axis and the coordinate of the weight- bearing line on a full-length standing anteroposterior radio- graph (3.9° ± 1.0° vs. 2.7° ± 2.2° of valgus, P = 0.01), (62.3 ± 2.9% vs. 58.7 ± 6.6% coordinate at the tibial plateau, P = 0.01).  - There was no significant difference in the alteration of tibial slope between the two groups.  - On clinical assessment, the navigation group showed better results in both the mean Hospital for Special Surgery knee score (84 ± 8 vs. 79 ± 7, P\0.01) and the mean Lysholm knee score (85 ± 6 vs. 83 ± 5, P \ 0.05).  - There was no significant difference in operation times between the two groups. | - Kinematic navigation-guided high tibial osteotomy is a reproducible and reliable procedure compared to conventional high tibial osteotomy. |
| Maurer et al. 2006 | - HTOs were performed with the open-wedge technique described by Staubli, using Tomofix stabilization aiming to achieve a postoperative genu valgum of 3° (2°-5°), meaning that the load line must be brought to the Fujisawa point in the lateral aspect of the joint compartment.   - In all cases, pre- operative planning was based on full-leg radiography and standard anterioposterior (AP)-view and lateral-view radiography, which included the drawing of the weight-bearing axis, the intended correction, and the opening wedge required for a 3° valgus. | - Good correlation was found not only between preoperative planning and preoperative computed navigation regarding the angle of varus deformity, but also between the navigated correction angle and postoperative measurements in full-leg view.  - Use of navigation resulted in fewer outliers and statistically significant higher accuracy of the postoperative leg axis within a range of 3° to 5° valgus. | - Navigation in HTO, which allows intraoperative calculation of the leg axis, seems to be a reliable and safe procedure, thus contributing to better clinical outcomes. |
| Saragaglia et al. 2005 | - Rigid body markers in navigated HTO were fixed percutaneously at the level of the distal femur and proximal tibia, allowing acquisition of the centers of the hip, knee, and ankle. The lower limb mechanical axis then appears on the screen and compared to the preoperative radiological goniometry.   - The HTO is performed 3 cm below the level of the medial joint line, and the level is confirmed by the placement of an intra-articular needle. The osteotomy is directed to the fibula head, keeping the saw horizontal to avoid fracturing the lateral tibial plateau. Using two Pauwels osteotomes the tibia is placed into valgus, and replaced by a metal spacer.  - If 8° of varus is present, then a surgeon uses 10-11mm spacer and ensure that an appropriate hypercorrection is produced in real-time on the screen. If the hypercorrection is insufficient, then a thicker spacer is used. Plating the proximal tibia completes the intervention. | - This study was divided into two sections, the first was a cohort study comparing navigated and conventional HTO. The results showed a 96% reproducibility in achieving a mechanical axis of 184° ± 2° in the navigated group compared to a 71% reproducibility in achieving a mechanical axis of 184° ± 2 ° in the conventional osteotomy group (P<0.0015).  - The second study was a prospective study on double level osteotomy and showed that the preoperative goal of 182° ± 2° has been achieved in 91% of patients. | - The navigated HTO for osteoarthritis secondary to genu varum is a reliable and reproducible technique that has helped the authors to achieve preoperative objectives in 96% of patients. The kinematic characteristics of OrthoPilot are well adapted to help surgeons perform osteotomies without arthrotomies in the majority of patients.   - Navigated double-level osteotomy is reliable, precise and reproducible for treating patients with excessive genu varum of the tibia and femur. |
| Ribeiro et al. 2014 | - Anatomic parameters of the hip, knee and ankle were marked for the navigation data collection.   - For the osteotomy, a longitudinal incision of 8–10 cm was made on the anteromedial border of the tibia. The pes anserinus tendons and superficial medial collateral ligament were dissected from the bone at the site of the osteotomy.   - Using a fluoroscope, a Kirshner guide wire was inserted, oriented medially to laterally, 1 cm from the articular surface of the tibia. Two more Kirschner wires were inserted, parallel with each other, oriented medially to laterally towards the head of the fibula.  - The osteotomy was then performed in the medial, anterior and posterior cortical bones, immediately below the Kirschner wires, using an oscillating saw. The osteotomy was completed using a chisel, under direct fluoroscopic visualization. | - The two groups were similar for preoperative mechanical axis (mean 8.10 ± 3.14 for the control and 6.60 ± 2.50 for the navigated group), pre-operative tibial slope (mean 8.95 ± 3.47 versus 8.17 ± 3.11, respectively) and Lyshom score (40.85 ± 15.46 and 44.83 ± 16.86).  - After surgery, the control group presented mean mechanical axis of (3.35 ± 3.27), tibial slope of (13.75 ± 3.75) and Lyshom score of (87.60 ± 11.12). The navigated group showed a postoperative mechanical axis mean of (3.06 ± 1.70), tibial slope of (10.11 ± 0.18) and Lyshom score of (91.94 ± 11.61). | - The navigation system allowed a significantly better control of tibial slope. Patients operated with the navigation system had significantly better Lysholm scores. |
| Gebhard et al. 2009 | - Registration automatically starts with defining the femoral head then determining essential kinematic and anatomical landmarks to calculate the center of the knee and ankle.   - After determining the alignment parameters, the amount of leg axis correction needed is calculated.  - After planning, two K-wires guide the saw in the predetermined direction. The planned depth of the cut is shown on the navigation screen.  - A spreading device (spreader, SynthesÓ) is used from the medial side to continuously correct the leg axis. The fixation is also under navigation control, and after correct leg alignment, the osteotomy is fixed with the Synthes TomofixTM plate. | - The majority of navigated HTO patients fell within the tolerated limit of ±3° for leg axis deviation, however, seven patients were reported with deviations outside of this range: three patients had deviations of > 3°–4.5° and four patients > 4.5°, respectively.  - Eight intraoperative complications were documented, partially resulting from technical problems associated with the navigation system.  - During the 6-week follow-up period, three postoperative complications were experienced, all not associated with navigation technology. | - In about 85% of cases, a perfect result in terms of deviation of the planned mechanical leg axis could be achieved. Computer assistance in HTO proved to be a helpful tool regarding intraoperative control of leg axis. |
| Lutzner et al. 2010 | - Navigation system OrthoPilot was used to measure limb alignment before and after surgery, through registering the mechanical axis in degrees varus or valgus mal-alignment and the intersection of the weight-bearing line with the tibial plateau.  - A medial opening osteotomy was performed in all cases and a spacer according to the preoperative planning was inserted.  - The leg alignment was controlled with fluoroscopic images of the hip, knee and ankle joint with the cable method. Then the plate was fixed with two cancellous screws proximally and two bicortical screws distally.  - The final analysis of the mechanical axis and the weight-bearing line was done using the navigation system. | - The mean deviation of the weight-bearing line from the aimed 80% intersection of the tibial plateau was 1.0% (0% to 2%) in the navigated and 8.6% (2% to 22.5%) in the conventional operated legs (p = 0.002).  - The postoperative weight-bearing line was in all navigated cases but only 5 of the 9 conventional operated cases (55%) within a ± 5% tolerance level from the desired 80% intersection of the tibial plateau (p = 0.33). | - Navigated open-wedge HTO achieved better correction of the weight-bearing line than the conventional method in human cadaver legs. |
| Hankemeier et al.2006 | - During navigated HTO, using the navigation module (Medivision), reference base was fixed at the distal femur to the proximal and distal tibia for landmark registration.  - The osteotomy height, length, and plane were continuously shown on the navigation monitor, and after incomplete osteotomy, gradual valgisation was performed under continuous assessment of the frontal, sagittal, and transverse planes.   - The intersection of the mechanical axis with the tibial plateau, MPTA, and mechanical femorotibial axis were measured, and after passing through 80% of the tibial plateau, the osteotomy was stabilized with a fixed angle implant with inter-locking screws to minimize postoperative loss of correction. | - The medial proximal tibia angle (MPTA) increased by 9.1±2.9° (range 5.2°–12.3°) on the average after navigated HTO and by 8.9±2.9° (range 4.7°–12.6°) after conventional HTO.   - After navigated HTO, the mechanical axis passed the tibial plateau through 79.7% (range 75.5–85.8%). Compared to conventional HTO, the average intersection of the mechanical axis was at 72.1% (range 60.4–82.4%) (P=0.020).   - The variability of the mean corrections was significantly lower in the navigated group (3.3% vs. 7.2%, P=0.012) | - Navigation systems provide intraoperative 3-dimensional real time control of the frontal, sagittal, and transverse axis and may increase accuracy of open-wedge HTO. |
| Lutzner et al. 2009 | - The OrthoPilot navigation system was used to measure limb alignment, as bicortical trackers were fixed at the distal femur und the tibia, thus permitting registration of kinematic and anatomical landmarks of the knee.  - After taking measurements with correct data acquisition, different landmarks were moved to obviously wrong places to study the influence of mistakes during landmark registration, such as:  (insufficient fixation of the pelvis during hip center acquisition, medialisation and lateralisation of the anatomic knee center of 15mm, ventralisation of the medial, the lateral and both epicondyles of 10mm, medialisation and lateralisation of the ankle center of 10mm. Only one landmark was moved at a time.   - After each move, the mechanical leg axis was recorded. | - The radiographic and direct measurement on the cadaver leg showed a mechanical leg axis of 4° varus malalignment.   - Lower limb alignment measurements with the navigation system yielded an overall mean mechanical leg axis of 3.9° ± 0.7° (2.7° to 5.1°) of varus malalignment.   - Calculated relative to 4° of varus malalignment obtained by radiography and controlled by direct measurement, the mean error was 0.6°. | - The computer-assisted navigation system provided precise information about the mechanical leg axis, irrespective of the observer’s experience. |
