# Supplementary material for: Navigation versus conventional high tibial osteotomy: systematic review
Source: Springerplus. 2015 Jun 17;4:271. doi: 10.1186/s40064-015-1040-5 (PMC4469593; doi:10.1186/s40064-015-1040-5)
Supplement: Additional file 3: — Table S3. Rating system for the hierarchy of evidence (Melnyk 2005). [file 40064_2015_1040_MOESM3_ESM.docx]

| **Table S3 Rating System for the Hierarchy of Evidence[25]** | |
| --- | --- |
| **Evidence Level** | **Type of Evidence** |
| Level I | Evidence from a systematic review of all relevant randomized controlled trials, or evidence-based clinical practice guidelines based on systematic reviews of randomized controlled trial. |
| Level II | Evidence obtained from at least one well-designed Randomized Controlled Trial (RCT) |
| Level III | Evidence obtained from well-designed controlled trials without randomization, quasi-experimental |
| Level IV | Evidence from well-designed case-control and cohort studies |
| Level V | Evidence from systematic reviews of descriptive and qualitative studies |
| Level VI | Evidence from a single descriptive or qualitative study |
| Level VII | Evidence from the opinion of authorities and/or reports of expert committees |
